# Positive association between constipation and mild cognitive impairment in elders: A cross-sectional study

**DOI:** 10.1097/MD.0000000000039943

**Published:** 2024-10-04

**Authors:** Kai-Yong Huang, Zhen-Zhen Yu, Jia-Jun Tu, Xian-Yan Tang, Jin-Meng Huang, Tian-Ming Lu, Yu-Qian Lu, Mei-Chun Huang, Jing Zhou, Andrea B. Maier, Kaisy Xinhong Ye, Zi Yang, Lei Feng, Guo-Dong Lu

**Affiliations:** a Department of Occupational and Environmental Health, School of Public Health, Guangxi Medical University, Nanning, Guangxi, China; b Guangxi Colleges and Universities Key Laboratory of Prevention and Control of Highly Prevalent Diseases, School of Public Health, Guangxi Medical University, Nanning, Guangxi, China; c Guangxi Key Laboratory of Environment and Health Research, School of Public Health, Guangxi Medical University, Nanning, Guangxi, China; d Department of Toxicology, School of Public Health, Guangxi Medical University, Nanning, Guangxi, China; e Department of Epidemiology and Health Statistics, School of Public Health, Guangxi Medical University, Nanning, Guangxi, China; f Educational Evaluation and Faculty Development Center, Guangxi Medical University, Nanning, Guangxi, China; g Department of Physiology, School of Basic Medical Sciences, Guangxi Medical University, Nanning, Guangxi, China; h Department of Human Movement Sciences, @AgeAmsterdam, Amsterdam Movement Sciences, Faculty of Behavioural and Movement Sciences, Vrije Universiteit Amsterdam, Amsterdam, Netherlands; i Healthy Longevity Translational Research Program, Yong Loo Lin School of Medicine, National University of Singapore, Singapore; j Centre for Healthy Longevity, National University Health System, Singapore; k Department of Psychological Medicine, Yong Loo Lin School of Medicine, National University of Singapore, Singapore; l School of the Public Health, Fudan University, Shanghai, China.

**Keywords:** cognitive function, cognitive impairment, fruit consumption, older adults, stroke, years of schooling

## Abstract

This study aimed to examine the association between constipation and mild cognitive impairment (MCI); and further elucidate the possible mechanisms involved. A cross-sectional study was conducted among community-dwelling elders (N = 789) in Nanning, China. Trained research staffs collected detailed information through questionnaires and physical examinations. A Bayesian network model was used to explore the hypothesized causal path. Synergistic effects of constipation with infrequent fruit consumption, inactive physical exercise, or history of stroke were observed in the risks of MCI occurrence. The Bayesian network model analyses showed 3 hypothesized causal-association paths leading to MCI occurrence. Among these, constipation, history of stroke, and years of schooling were directly related to the occurrence of MCI. Years of schooling indirectly affected MCI through infrequent fruit consumption and constipation; or through inactive physical exercises and history of stroke. This study demonstrates a direct association between constipation and increased risks of MCI.

## 1. Introduction

With the acceleration of world’s population aging, the number of people living with dementia is rapidly growing from 55 million in 2019 to 139 million in 2050 worldwide, according to the latest World Health Organization global status report.^[1]^ Alzheimer disease (AD), the most common form of dementia, is a progressive neurodegenerative disease that causes memory and cognitive decline, accompanied by psychiatric symptoms and behavioral disorders.^[2]^ So far, there is few clinically-approved effective drug to treat AD (e.g. donepezil, rivastigmine, galantamine, memantine, and memantine combined with donepezil). Mild cognitive impairment (MCI), as an intermediate stage of cognitive impairment between physiological cognitive aging and pathological dementia, does not significantly affect daily life.^[3,4]^ A large number of population-based epidemiological investigations have reported that the prevalence of MCI ranged from 3% to 19% among people aged 65 and above.^[4,5]^ Around 30% to 50% of older adults with MCI progress to dementia within 5 years, compared with 3% of those without MCI at the same age.^[4–6]^ But some persons with MCI can remain stable or return to normal over time. That is why MCI can be regarded as a transition stage before the onset of dementia. However, it is yet to be validated whether MCI is a critical stage to prevent or delay the development of dementia by adopting the beneficial factors and/or controlling risk factors of MCI.

Constipation is a common health problem, especially among middle-aged and older people. Previous studies have shown that constipation is closely related to Parkinson disease (PD).^[7,8]^ Constipation can precede clinical motor symptoms of PD by many years.^[9]^ However, only a few studies associated constipation with cognitive function and cognitive diseases. Our previous study in 2020 first reported that infrequent bowel movement was associated with increased risk of MCI in community-living Singaporean older adults in a cross-sectional study.^[10]^ Later in the year of 2022, 3 studies consecutively reported the correlations of constipation with MCI, dementia or AD.^[11–13]^ Although these studies established the positive associations between constipation and cognitive declines, it is yet unclear how constipation promotes cognitive declines during aging. Accumulating evidence highlights the link between constipation and alteration of gut microbiome as a causative factor of mental diseases. Alterations in the structure and functions of gut microbiota may lead to the changes in brain function and behavior, a phenomenon so called microbe–gut–brain axis. This axis may provide a novel research angle to understand the development of mental diseases.

Therefore in the present study, we first determined the correlation between constipation and MCI in a cross-sectional study of adults aged 60 or older in Nanning, China. The second aim was to dissect how constipation affects MCI by analyzing the structure of the hypothesized causal associations and the inter-plays between constipation and other influencing factors of MCI.

## 2. Materials and methods

### 2.1. Participants and criteria

This population-based cross-sectional study recruited community-living residents (aged ≥ 60) in Nanning, the capital city of Guangxi province in China using a convenience sampling design in 2021. Well-trained research staffs conducted semi-structured interviews with participants and their caregivers in community health service centers or at their residence. For some contents that cannot accurately remember or report, we asked the older adults’ family members to help them remember or provide accurate answers. For disease history, medication history, and health history, we obtained accurate information by reviewing the older adults’ medical records and health files. The inclusive criteria for this study were community residents who were: (1) aged 60 years or older, (2) local resident for at least 1 year, (3) voluntary and cooperative for the survey. The exclusion criteria were those who: (1) were unable to conduct meaningful face-to-face interviews or complete all questionnaires and evaluations, (2) had been diagnosed with dementia or other major mental diseases (including PD, any acute phase of brain infectious diseases). Finally, a total of 789 participants were included in the study (Table 1). The response rate was 88.1%.

**Table 1:** Constipation and some socio-demographic factors were associated with MCI.

Variables	Control N = 658 n (%)	MCI N = 131 n (%)	*F*/*χ*^*2*^	*P*
Gender			5.849	.016
Male	286 (43.5)	42 (32.1)		
Female	372 (56.5)	89 (67.9)		
Age (mean ± SD)	69.9 ± 6.8	69.6 ± 7.4	0.199	.655
Years of schooling (mean ± SD)	7.9 ± 4.1	6.0 ± 4.6	20.993	<.001
Constipation			15.677	<.001
Yes	97 (14.7)	38 (29.0)		
No	561 (85.3)	93 (71.0)		
Ethnic*			5.052	.080
Han	419 (63.8)	70 (53.5)		
Zhuang	221 (33.6)	56 (42.7)		
Others	17 (2.6)	5 (3.8)		
Marital status			9.594	.002
Married/cohabitation	559 (85.0)	97 (74.0)		
Divorced/widowed/single	99 (15.0)	34 (26.0)		
Work status			0.044	.834
Still working	33 (5.0)	6 (4.6)		
Not working	625 (95.0)	125 (95.4)		
Annual family income*			4.572	.102
<30,000 RMB	308 (46.9)	74 (56.5)		
30,000–60,000 RMB	215 (32.7)	32 (24.4)		
>60,000 RMB	134 (20.4)	25 (19.1)		
Co-living situation			0.039	.843
Live alone	47 (7.1)	10 (7.6)		
Not live alone	611 (92.9)	121 (92.4)		

MCI = mild cognitive impairment, SD = standard deviation.

* Due to the missing values in some variables, the total numbers may not equal to 658 or 131.

### 2.2. Variables

Well-trained research staffs collected detailed information through questionnaire survey, including socio-demographic characteristics (age, gender, race, profession, degree of education, annual household income, marital status, and co-living situation), lifestyle and behavior (drinking, smoking, physical exercise, social activities, daytime outdoor activities, mental activities, indoor activities, sleep quality, and post-lunch nap status), medical comorbidities, and dietary habits, as described before.^[10]^

### 2.3. Neurocognitive assessment and constipation diagnosis

Cognition function was assessed using the Chinese version of Mini-Mental State Examination (MMSE). The total scores of the MMSE ranged from 0 to 30, with higher scores demonstrating better cognitive function.^[10]^ When the MMSE total scores were less than or equal to the cutoff (17 for illiterate participants, 20 for those with primary school education level, and 24 for individuals with secondary school and above),^[14]^ a further assessment were carried out, including Activity of Daily Living Scale^[15]^ for social functioning assessment, Clinical Dementia Rating^[16]^ for severity of dementia and cognitive decline assessment. The diagnosis of MCI was made via the expert panel consisting of 2 neurologists and neuropsychologists, and other clinical evaluation specialists with expertise in cognitive impairment, according to the diagnostic criteria proposed by Peterson.^[17]^

Constipation was assessed using a questionnaire according to the Rome Criteria IV. Criteria fulfilled for the last 3 months with symptom onset at least 6 months prior to diagnosis: (1) must include 2 or more of the following: I. straining during more than one-fourth (25%) of defecation; II. lumpy or hard stools (BSFS 1–2) more than one-fourth (25%) of defecation; III. sensation of incomplete evacuation more than one-fourth (25%) of defecation; IV. sensation of anorectal obstruction/blockage more than one-fourth (25%) of defecation; V. manual maneuvers to facilitate more than one-fourth (25%) of defecation (e.g., digital evacuation, support of the pelvic floor); VI. fewer than 3 spontaneous bowel movements per week. (2) Loose stools are rarely present without the use of laxatives. (3) Insufficient criteria for irritable bowel syndrome with constipation.^[18]^

### 2.4. Statistical analyses

Categorical variables were described by numbers and frequency, while continuous variables were described by mean and standard deviation. The data were analyzed using the Statistical Package for the Social Sciences 22.0 (IBM Corp., Armonk, NY). The chi-square tests were used to determine significant differences for categorical variables, while one-way analysis of variance were used to evaluate between-group differences for the continuous variables. A two-sided *P*-value <.05 was considered statistically significant.

To further determine the associations between constipation and MCI occurrence, multivariable logistic regression models was performed. The models were adjusted for gender, age, marital status, work status, smoking, and drinking. Those factors with statistical differences *P* < .1 under the chi-square test and one-way analysis of variance were included in the multivariable logistic regression models. After identifying the important influence factors of MCI, we explored interactions between constipation with these important influencing factors using the Statistical Package for the Social Sciences 22.0. Lastly, a Bayesian network model was used to reveal the hypothesized causal path between MCI occurrence and the significant factors, as described previously.^[10]^

After identifying the significant factors underlying the status of MCI, a Bayesian network model, structural equation model, and directed acyclic graph approach were integrated to reveal the structure of hypothesized causal associations between significant factors and MCI. Meanwhile, the probability inferences of the Bayesian network for the influencing factors of MCI were calculated, and the importance of each significant factor was quantitatively measured based on the Tree Augmented Naïve Bayesian network learning. The Bayesian network model was fitted with the bnlearn package via R software 3.5, and the Bayesian network-based causal relationships between exposure variables and MCI were visualized using the R package “dagitty.”^[10]^

### 2.5. Ethical approval

The study was approved by the Medical Ethics Committee of Guangxi Medical University (protocol number 20210132). All methods were carried out in accordance with relevant guidelines and regulations. Informed consent was obtained from all participants prior to their participation.

## 3. Results

### 3.1. Constipation and year of schooling were associated with MCI

Among the participated 789 older adults, 131 (16.6%) were diagnosed with MCI (MCI group) and 658 (83.4%) with normal cognitive function (control group) by the expert panels (Table 1). The MCI older adults had higher proportions of females (67.9% vs 56.5%, *P* = .016) and constipation (29.0% vs 14.7%, *P* < .001) but shorter years of schooling (6.0 ± 4.6 vs 7.9 ± 4.1 years, *P* < .001) than the control peers. Although few older adults (7.6% and 7.1%) lived alone, a higher proportion of older adults in the MCI group got divorced/widowed or kept single, compared with those in the control group (25.0% vs 15.0%, *P* = .002). Besides, there were no statistical differences between the 2 groups in age, ethnicity, current work status, and annual family income (all *P* > .05).

### 3.2. Other statistically important influencing factors of MCI

As shown in Table 2 of medical comorbidities, the proportions of history of stroke (13.0% vs 4.9%, *P* < .001), history of head trauma (3.1% vs 0.3%, *P* = .006) are statistically higher while hypertension (39.7% vs 31.2%, *P* = .057) tend to be higher in the MCI group than those in the control group, respectively. In contrast, other medical comorbidities did not differ in the 2 groups (Table S1, Supplemental Digital Content, http://links.lww.com/MD/N670). Among the common serum variables (Table 2 and Table S2, Supplemental Digital Content, http://links.lww.com/MD/N670), the concentrations of aspartate aminotransferase (23.58 ± 10.37 vs 21.89 ± 8.43 U/L, *P* = .045), and low density lipoprotein cholesterol (LDL-C) (3.23 ± 0.89 vs 2.74 ± 0.82 mmol/L, *P* < .001) were higher, but creatinine (86.29 ± 23.83 vs 80.52 ± 22.85 μmol/L, *P* = .011) lower, in the MCI group than in the control group. Furthermore among all the surveyed lifestyle factors (Table 2 and Table S3, Supplemental Digital Content, http://links.lww.com/MD/N670), more participants in the MCI group had infrequent (≤3 per week) physical exercise (48.9% vs 21.6%, *P* < .001), social activities (55.0% vs 39.7%, *P* = .001) or daytime outdoor activities (36.6% vs 19.9%, *P* < .001). In line of these inactive lifestyles, more participants in the MCI group had infrequent fruit consumption (≤3 days per week, 36.6% vs 22.2%, *P* < .001) (Table 2 and Table S4, Supplemental Digital Content, http://links.lww.com/MD/N670).

**Table 2:** Other statistically important influential factors of MCI.

Variables	Control N = 658 n (%)	MCI N = 131 n (%)	*χ* ^*2*^	*P*
Hypertension			3.628	.057
Yes	205 (31.2)	52 (39.7)		
No	453 (68.8)	79 (60.3)		
History of stroke			12.348	<.001
Yes	32 (4.9)	17 (13.0)		
No	626 (95.1)	114 (87.0)		
History of head trauma			7.604	.006
Yes	2 (0.3)	4 (3.1)		
No	656 (99.7)	127 (96.9)		
How often participate in physical exercise			42.126	<.001
≤3 per week	142 (21.6)	64 (48.9)		
≥4 per week	516 (78.4)	67 (51.1)		
How often participate in social activities			10.479	.001
≤3 per week	261 (39.7)	72 (55.0)		
≥4 per week	397 (60.3)	59 (45.0)		
How often participate in outdoor activities in daytime			17.438	<.001
≤3 per week	131 (19.9)	48 (36.6)		
≥4 per week	527 (80.1)	83 (63.4)		
How often consume fruits			12.307	<.001
≤3 per week	146 (22.2)	48 (36.6)		
≥4 per week	512 (77.8)	83 (63.4)		
Waistline (cm) (mean ± SD)	85.56 ± 9.11	87.18 ± 9.22	3.473	.063
AST (U/L) (mean ± SD)	21.89 ± 8.43	23.58 ± 10.37	4.038	.045
Creatinine (μmol/L) (mean ± SD)	86.29 ± 23.83	80.52 ± 22.85	6.494	.011
LDL-C (mmol/L) (mean ± SD)	2.74 ± 0.82	3.23 ± 0.89	38.205	<.001

AST = aspartate aminotransferase, LDL-C = low density lipoprotein cholesterol, MCI = mild cognitive impairment, SD = standard deviation.

### 3.3. Constipation was independently associated with MCI by multivariate logistic regression analysis

It was found that more years of schooling was associated with decreased risk of MCI occurrence (adjusted odds ratios [AOR] = 0.929, 95% confidence interval [CI] 0.882–0.979, *P* = .006), after adjusting gender, age, marital status, work status, smoking, and drinking. In contrast, constipation (AOR = 1.937, 95% CI 1.178–3.186, *P* = .009), history of stroke (AOR = 2.719, 95% CI 1.307–5.654, *P* = .007), history of head trauma (AOR = 7.996, 95% CI 1.139–56.151, *P* = .037), inactive physical exercise (AOR = 2.641, 95% CI 1.698–4.108, *P* < .001), inactive social activities (AOR = 1.632, 95% CI 1.063–2.508, *P* = .025), infrequent fruits consumption (AOR = 1.692, 95% CI 1.067–2.685, *P* = .025), and LDL-C (AOR = 2.042, 95% CI 1.578–2.641, *P* < .001) were statistically associated with increased risks of MCI in older adults (Table 3).

**Table 3:** Multivariate analysis for MCI influential factors.

Variables	*β*	Wald	*P*	AOR	95% *CI*
Constipation	0.661	6.791	.009	1.937	1.178–3.186
Years of schooling	−0.073	7.516	.006	0.929	0.882–0.979
Inactive physical exercise	0.971	18.573	<.001	2.641	1.698–4.108
Inactive social activity	0.490	5.003	.025	1.632	1.063–2.508
History of stroke	1.000	7.165	.007	2.719	1.307–5.654
History of head trauma	2.079	4.370	.037	7.996	1.139–56.151
Infrequent fruit consumption	0.526	4.990	.025	1.692	1.067–2.685
LDL-C (mmol/L)	0.714	29.554	<.001	2.042	1.578–2.641

Model was adjusted for gender, age, marital status, work status, smoking, and drinking.

AOR = adjusted odds ratios, CI = confidence interval, LDL-C = low density lipoprotein cholesterol.

### 3.4 . Interactions between constipation with other important influencing factors

**Figure 1.**
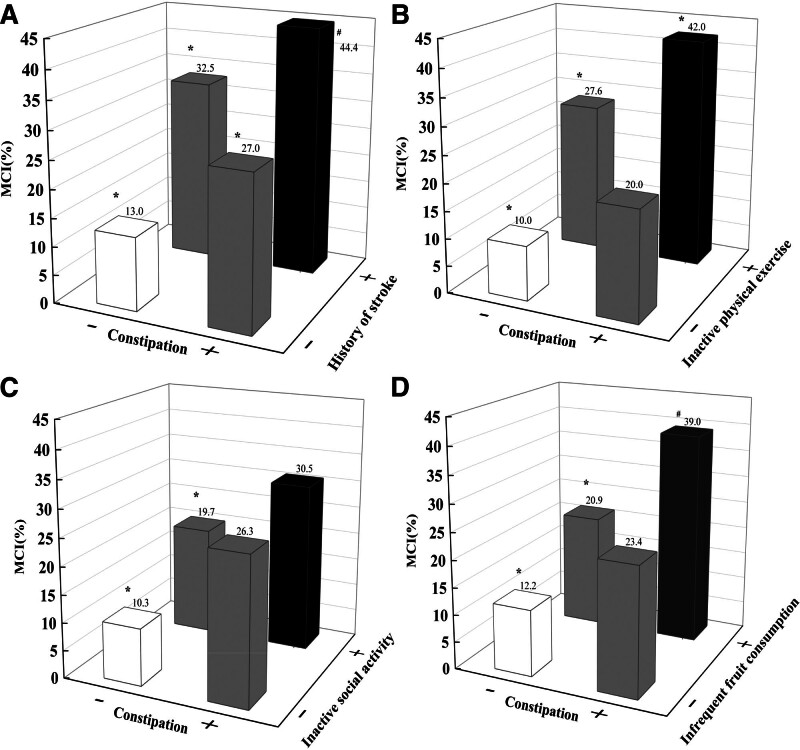
The respective interactions of constipation with other important influencing factors. The respective interactions of constipation with other important influencing factors included history of stroke (A), inactive physical exercise (B), inactive social activities (C), and infrequent fruit consumption (D). *P < .01, ^#^*P* < .05.

The next question to explore was whether constipation can synergize with other independent influencing factors to affect MCI. We then explored their interactions. As shown in Table 4, constipation can combine with years of schooling, inactive physical exercise, inactive social activity, history of stroke, history of head trauma, and infrequent fruit consumption to affect more MCI (*P* < .001 for all). Particularly, the combination of constipation with inactive physical exercise affected a higher proportion of MCI than simple addition of the effects of the 2 factors alone (32.0% vs [17.6% + 10.0%], Table 5 and Fig. 1). Similar results was observed in the combination of constipation with infrequent fruit consumption (26.8% vs [8.7% + 11.2%], Table 5 and Fig. 1).

**Table 4:** Interactions between constipation with other important influential factors.

Variables	*P*	OR	95% CI
Constipation × years of schooling	<.001	1.604	1.038–1.090
Constipation × inactive physical exercise	<.001	1.790	1.509–2.123
Constipation × inactive social activity	<.001	1.547	1.294–1.850
Constipation × history of stroke	<.001	1.641	1.353–1.991
Constipation × history of head trauma	<.001	1.610	1.305–1.987
Constipation × infrequent fruit consumption	<.001	1.515	1.277–1.797

CI = confidence interval, OR = odds ratios.

**Table 5:** Stratification analysis between constipation with other important influential factors.

Constipation	History of stroke	Control	MCI	*P*	OR	95% CI
No	No	534 (87.0)	80 (13.0)	<.001		
No	Yes	27 (67.5)	13 (32.5)	.001	0.311	0.154–0.628
Yes	No	92 (73.0)	34 (27.0)	<.001	0.405	0.256–0.641
Yes	Yes	5 (55.6)	4 (44.4)	.014	0.187	0.049–0.712
Constipation	Inactive physical exercise					
No	No	448 (90.0)	50 (10.0)	<.001		
No	Yes	113 (72.4)	43 (27.6)	<.001	6.488	3.445–12.220
Yes	No	68 (80.0)	17 (20.0)	.057	1.903	0.981–3.691
Yes	Yes	29 (58.0)	21 (42.0)	.007	2.897	1.337–6.276
Constipation	Inactive social activity					
No	No	341 (89.7)	39 (10.3)	<.001		
No	Yes	220 (80.3)	54 (19.7)	<.001	3.839	2.013–7.321
Yes	No	56 (73.7)	20 (26.3)	.070	1.789	0.954–3.355
Yes	Yes	41 (69.5)	18 (30.5)	.591	1.229	0.579–2.612
Constipation	Infrequent fruit consumption					
No	No	440 (87.8)	61 (12.2)	<.001		
No	Yes	121 (79.1)	32 (20.9)	.007	1.908	1.189–3.061
Yes	No	72 (76.6)	22 (23.4)	.078	1.703	0.942–3.080
Yes	Yes	25 (61.0)	16 (39.0)	<.001	6.059	3.189–11.479

CI = confidence interval, MCI = mild cognitive impairment, OR = odds ratios.

### 3.5. Bayesian network model analysis of the causal-association diagram of MCI

**Figure 2.**
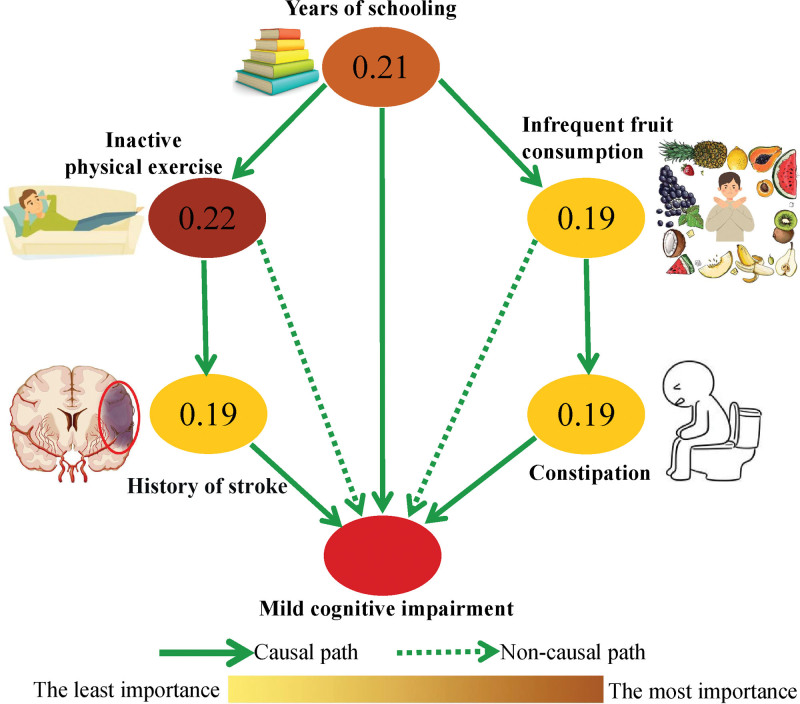
Bayesian network causal inference of MCI and influencing factors. Bayesian network approach explored the causal-association diagram between MCI and exposure variables, and the importance of exposure variables. The higher magnitude the value is, more important the variable. Meanwhile, the importance of variables was visualized via the gradual changes of color of nodes.

Finally, a Bayesian network model was used to explore the hypothesized causal-association diagram between MCI occurrence and independent influencing factors found in the multivariate logistic regression analysis (Table 3). As shown in Figure 2, the results indicated 3 hypothesized causal-association paths leading to MCI occurrence. Among these, constipation, history of stroke, and years of schooling were directly related to the occurrence of MCI. Years of schooling might cause MCI indirectly through infrequent fruit consumption then constipation. In addition, years of schooling could also result in MCI indirectly through inactive physical exercise then history of stroke. The Bayesian network model causal path analysis further estimated the score of importance of the significant influence factors. Inactive physical exercise was regarded as the most important factor with an inferred score of 0.22, followed by years of schooling (0.21), constipation (0.19), infrequent fruit consumption (0.19), and history of stroke (0.19).

## 4. Discussion

The present study clearly revealed that constipation was independently associated with the increased risks of MCI occurrence among older adults in Nanning, China. Constipation could combine with inactive physical exercise or infrequent fruit consumption to affect more MCI patients. More importantly, we demonstrated that constipation, together with years of schooling and history of stroke, may directly cause MCI through the analysis of Bayesian network model. Years of schooling may further indirectly affect MCI through infrequent fruit consumption and then constipation.

We previously reported that inactive bowel movement was associated with MCI in a community-living Singaporean cohort.^[10]^ The study was conducted by a survey including bowel movement frequencies, which might be limited by lack of clinical diagnosis and subjective self-report. Thus in the present study, we applied Rome Criteria IV to evaluate constipation. Beside our initial MCI report of inactive bowel movement, 3 other studies recently confirmed the association of constipation with cognitive diseases including MCI, dementia or AD.^[11–13]^ Furthermore, Leta and coauthors reported that constipation was an independent predictor of dementia in 2 independent cohorts of patients with de novo PD (hazard ratio = 2.311, *P* = .02).^[19]^ The PD patients with constipation of the 2 cohorts also had a faster progression speed to develop dementia.

However, how constipation affects MCI and other cognitive diseases is yet unclear. Our present study suggested that beside the direct effect of constipation, years of schooling, and infrequent fruit consumption, 2 common influencing factors found in our cross-sectional Singaporean study,^[10]^ may sequentially act upstream of constipation (Fig. 2). First, it is well known that higher educational level, which is almost equivalent to longer education years, results in more cognitive reserve.^[3,6]^ Childhood education, as an important modifiable beneficial factor for prevention of AD or MCI, was thus highly recommended as a measure with Class I (strong recommendation) in the Guideline for prevention of AD.^[20]^ Second, higher education level was independently associated with higher consumption of fresh fruits,^[15,21]^ which was in line with the current study. It has long been recognized that fruits positively affected intestinal function. Rich in dietary fiber, fruits play important roles in promoting intestinal function via increasing stool volume, improving stool consistency, and facilitating a healthy microbiota ecosystem.^[22]^ But it is yet not clear why consumption of vegetables which are also rich in dietary fiber and vitamins was not associated with MCI in both the present Nanning cohort and our previous Singaporean cohort.

Constipation is closely related to alteration of gut microbiome. Furthermore, alteration of gut microbiome was able to trigger inflammatory response, and then affected brain function and behavior, ultimately lead to a range of central nervous system diseases such as PD, AD, cognitive disorders, depression, and autism spectrum disorders.^[13,23,24]^ Neurotransmitters and their precursors produced and released by the gut microbiota may also participate in mental diseases.^[24]^ For instance, *Clostridium sporogenes* and *Ruminococcus gnavus* from phylum Firmicutes in the human intestine were identified to influence host behavior by regulating the metabolism of tryptophan and serotonin in the brain and peripheral system.^[25]^ Thus, it is possible that constipation might affect MCI through alteration of gut microbiota.

The current study also found that history of stroke was directly associated with increased risks of MCI occurrence among older adults. Furthermore, years of schooling and inactive physical exercises may sequentially reside upstream of stroke (Fig. 2). First, stroke not only contributes to vascular dementia, but also occurs more commonly in older adults with AD than those without.^[6]^ The population-based Oxford Vascular Study demonstrated that the patients with stroke had 50 times higher risk in developing dementia compared with those without stroke.^[26]^ Second, many studies pointed out that physical activity and exercise could prevent stroke through modifying stroke risk factors.^[27]^ Physical exercise could enhance the expression of brain derived neurotrophic factor in peripheral blood, which played an important role in the genesis and development of nerve cells, synaptic plasticity, survival and repair of nerve cells.^[28]^ A systematic review with meta-analysis showed similar results that mind-body exercise (tai chi, yoga, qigong) had the potential to improve various cognitive functions in people with MCI.^[29]^ Third, people with higher education level may pay more attention to physical health, learn more health promotion methods, and then participate in physical exercise more actively.^[30]^ That is why physical exercise was also highly recommended as a measure with Class I (strong recommendation), level B in the Guideline for prevention of AD.^[20]^

Active engagement in social activities may be potentially protective against progression to MCI or delaying further cognitive decline among older adults.^[6,31]^ Consistent to our results of inactive social activities, a great number of studies indicated that people with little social activity engagement and infrequent social contact might have an increased risk of all-cause dementia.^[6]^ Again, social activities, was a beneficial factor being recommended as a measure with level C in the Guideline for prevention of AD.^[20]^

This study also revealed an association between history of head trauma and increased risks of MCI. In recent years, some studies showed higher prevalence of self-reported head trauma history in patients with MCI and AD.^[6,32,33]^ Mild traumatic brain injury might contribute to acceleration of cognitive decline, as an independent pathogenic factor to intensify neurodegenerative mechanisms of AD.^[33,34]^ Therefore, active prevention or management of head injuries should be considered to maintain cognitive function in older adults.

Lastly, the present study found that LDL-C was markedly associated with increased risks of MCI among older adults, suggesting that the elevated LDL-C levels might be a potential risk factor for MCI.^[35]^ A longitudinal study of Chinese older adults found that higher blood concentrations of LDL-C in late-life was associated with faster cognitive decline.^[36]^ The concentration of LDL-C was correlated with the incidence and severity of coronary heart disease. Another study reported that LDL-C, together with coronary heart disease, hypertension, and total cholesterol, were independent risk factors for MCI.^[37]^

Although this study revealed the lack of significant associations between certain factors (BMI, hypertension, diabetes, and heart disease) and MCI, some studies found that these factors were important influencing factors for MCI. The reason for the inconsistent research results may be that the present study was conducted in a single city, which may not be representative of the broader population. Moreover, there were significant differences in lifestyle, economic culture, and dietary habits among populations in different regions. Therefore, it is necessary to conduct a multi-center cohort study to obtain more comprehensive, accurate, and reliable results. A large sample study in China showed that dementia and MCI shared similar risk factors including hypertension, hyperlipidemia, diabetes, heart disease, and cerebrovascular disease.^[3]^ Additionally, our previous study in Singaporean also indicated hypertension was positively associated with MCI occurrence.^[10]^ Therefore, the Guideline for prevention of AD highly recommends that individuals aged <65 years should avoid hypertension via a healthier lifestyle.^[20]^ In recent years, a cohort study suggested that among cognitively intact people, significantly lower BMI occurs beginning approximately 7 years before MCI diagnosis. After MCI diagnosis, BMI declined at the same pace in people who developed dementia and those who did not.^[38]^ The Guideline for prevention of AD also highly recommends that adults aged <65 years should maintain/achieve a BMI between 18.5 and 24.9 kg/m^2^, and adults aged >65 years should not to be too skinny.^[20]^ These findings highlight the importance of monitoring weight change regularly among older adults.

This study explored a novel influence factor (constipation) on MCI, and analyzed the interactions between constipation with other important influencing factors, and then a Bayesian network model was used to reveal the hypothesized causal path between MCI and the significant influencing factors. The causal path of the influencing factors and MCI were well explained by the Bayesian network model.

Our study has several limitations that have to be acknowledged. First, it is a cross-sectional study that recruited older adults from Nanning, a Southern city of China. This one-city study may not be fully representative of the total population. Second, because many data were collected based on self-report survey from older adults, recalling bias is unavoidable. We thus excluded all dementia patients to minimize the recalling bias. Third, although the diagnosis of constipation was based on the Rome Criteria IV, there is still lack of objective indicators for evaluation.

## 5. Conclusions

The present study demonstrated that constipation, together with history of stroke, history of head trauma, inactive physical exercise, inactive social activities, infrequency fruit consumption, and LDL-C were closely associated with increased risks of MCI in older adults. In contrast, more years of schooling was associated with decreased risk of MCI. The Bayesian network model analysis further revealed 3 hypothesized causal-association paths leading to MCI occurrence. Among these, constipation, history of stroke and years of schooling were directly related to the occurrence of MCI. Years of schooling might also affect MCI indirectly sequentially through fruit consumption and constipation, or sequentially through inactive physical exercise and stroke. These findings might provide a new direction for the study of the pathogenesis of MCI and a new theoretical basis for the prevention of MCI. We recommend that the health authority conduct a comprehensive assessment of the factors affecting MCI, so as to guide the adoption of measures to prevent MCI. Therefore, active control and reduction risk factors of MCI should be considered to maintain cognitive function and prevent MCI in older adults.

## Acknowledgments

The authors are grateful to the participants of this study. The authors also extend their appreciation to the Medical Ethics Committee of Guangxi Medical University, for giving the approval for this study.

## Author contributions

**Conceptualization:** Lei Feng, Guo-Dong Lu.

**Data curation:** Jin-Meng Huang, Jing Zhou, Andrea B Maier, Kaisy Xinhong Ye, Lei Feng.

**Formal analysis:** Kai-Yong Huang, Zhen-Zhen Yu, Jia-Jun Tu, Xian-Yan Tang.

**Funding acquisition:** Kai-Yong Huang, Jing Zhou, Zi Yang, Guo-Dong Lu.

**Investigation:** Tian-Ming Lu, Yu-Qian Lu, Mei-Chun Huang, Zi Yang.

**Methodology:** Kai-Yong Huang, Zhen-Zhen Yu.

**Software:** Jia-Jun Tu, Xian-Yan Tang, Mei-Chun Huang, Zi Yang.

**Validation:** Jin-Meng Huang, Jing Zhou, Andrea B Maier.

**Visualization:** Jin-Meng Huang, Tian-Ming Lu, Yu-Qian Lu, Kaisy Xinhong Ye.

**Writing – original draft:** Kai-Yong Huang, Zhen-Zhen Yu, Jia-Jun Tu, Xian-Yan Tang.

**Writing – review & editing:** Lei Feng, Guo-Dong Lu.

## Supplementary Material


