# Supplementary material for: Positive association between constipation and mild cognitive impairment in elders: A cross-sectional study
Source: Medicine (Baltimore). 2024 Oct 4;103(40):e39943. doi: 10.1097/MD.0000000000039943 (PMC11460931; doi:10.1097/MD.0000000000039943)
Supplement: Supplementary file 1 [file medi-103-e39943-s001.doc]

**Supplementary Table 1. Other medical comorbidities between MCI elders and controls [n (%)]**

| **Variables** | **Control**  **N=658**  **n (%)** | **MCI**  **N=131**  **n (%)** | ***χ^2^*** | ***P*** |
| --- | --- | --- | --- | --- |
| **Hyperlipidemia** |  |  | 1.408 | .235 |
| Yes | 79 (12.0) | 11 (8.4) |  |  |
| No | 579 (88.0) | 120 (91.6) |  |  |
| **Heart failure** # |  |  | - | 1.000 |
| Yes | 1 (0.2) | 0 (0.0) |  |  |
| No | 657 (99.8) | 131 (100.0) |  |  |
| **Heart attack** |  |  | 0.268 | .605 |
| Yes | 32 (4.9) | 5 (3.8) |  |  |
| No | 626 (95.1) | 126 (96.2) |  |  |
| **Arrhythmia** |  |  | 0.098 | .754 |
| Yes | 16 (2.4) | 2 (1.5) |  |  |
| No | 642 (97.6) | 129 (98.5) |  |  |
| **Asthma** # |  |  | - | .597 |
| Yes | 5 (0.8) | 0 (0.0) |  |  |
| No | 653 (99.2) | 131 (100.0) |  |  |
| **Chronic bronchitis / Emphysema** # |  |  | - | .143 |
| Yes | 14 (2.1) | 0 (0.0) |  |  |
| No | 644 (97.9) | 131 (100.0) |  |  |
| **Chronic obstructive pulmonary disease** # |  |  | - | .607 |
| Yes | 7 (1.1) | 0 (0.0) |  |  |
| No | 651 (98.9) | 131 (100.0) |  |  |
| **Tuberculosis** |  |  | <0.001 | 1.000 |
| Yes | 4 (0.6) | 1 (0.8) |  |  |
| No | 654 (99.4) | 130 (99.2) |  |  |
| **Deafness** |  |  | <0.001 | 1.000 |
| Yes | 18 (2.7) | 3 (2.3) |  |  |
| No | 640 (97.3) | 128 (97.7) |  |  |
| **Cataracts/glaucoma** |  |  | 0.309 | .578 |
| Yes | 37 (5.6) | 9 (6.9) |  |  |
| No | 621 (94.4) | 122 (93.1) |  |  |
| **Chronic kidney disease** |  |  | 0.821 | .365 |
| Yes | 6 (0.9) | 3 (2.3) |  |  |
| No | 652 (99.1) | 128 (97.7) |  |  |
| **Arthrophlogosis** |  |  | 0.002 | .968 |
| Yes | 61 (9.3) | 12 (9.2) |  |  |
| No | 597 (90.7) | 119 (90.8) |  |  |
| **Osteoporosis** |  |  | 0.093 | .760 |
| Yes | 45 (6.8) | 8 (6.1) |  |  |
| No | 613 (93.2) | 123 (93.9) |  |  |
| **Gastrointestinal problems** |  |  | 0.784 | .376 |
| Yes | 78 (11.9) | 12 (9.2) |  |  |
| No | 580 (88.1) | 119 (90.8) |  |  |
| **Thyroid disease** |  |  | 0.019 | .891 |
| Yes | 9 (1.4) | 1 (0.8) |  |  |
| No | 649 (98.6) | 130 (99.2) |  |  |
| **Cancer** |  |  | <0.001 | 1.000 |
| Yes | 8 (1.2) | 2 (1.5) |  |  |
| No | 650 (98.8) | 129 (98.5) |  |  |
| **Nervous system disease** |  |  | 0.119 | .730 |
| Yes | 5 (0.8) | 2 (1.5) |  |  |
| No | 653 (99.2) | 129 (98.5) |  |  |
| **Mental disease** |  |  | <0.001 | 1.000 |
| Yes | 3 (0.5) | 1 (0.8) |  |  |
| No | 655 (99.5) | 130 (99.2) |  |  |

# Fisher’s exact test. MCI, mild cognitive impairment.

**Supplementary Table 2. Other Physical Examination Item between MCI and Control E**lders (Mean±SD)

| **Variables** | **Control**  **N=658**  **n (%)** | **MCI**  **N=131**  **n (%)** | ***F*** | ***P*** |
| --- | --- | --- | --- | --- |
| BMI (kg/m^2^) | 23.42±3.07 | 23.77±3.16 | 1.437 | .231 |
| WBC (10^9^/L) | 6.28±1.60 | 6.12±1.57 | 1.063 | .303 |
| RBC (10^12^/L) | 4.57±0.61 | 4.49±0.58 | 2.051 | .153 |
| Hemoglobin (g/L) | 132.61±16.25 | 131.42±17.11 | 0.574 | .449 |
| Blood platelet (10^9^/L) | 243.36±72.85 | 232.15±56.79 | 2.763 | .097 |
| TBil (μ mol/L) | 13.19±4.88 | 12.88±5.22 | 0.432 | .511 |
| ALT (U/L) | 18.76±12.33 | 19.71±11.13 | 0.658 | .418 |
| BUN (mmol/L) | 5.72±1.83 | 5.73±1.85 | 0.008 | .931 |
| Uric acid (μmol/L) | 364.71±95.29 | 361.20±93.92 | 0.149 | .699 |
| TC (mmol/L) | 5.26±1.25 | 5.33±1.12 | 0.420 | .517 |
| TG (mmol/L) | 1.58±1.14 | 1.74±1.44 | 2.065 | .151 |
| HDL-C (mmol/L) | 1.45±0.38 | 1.43±0.36 | 0.220 | .639 |
| GLU (mmol/L) | 6.01±3.22 | 5.72±1.60 | 1.005 | .316 |

MCI, mild cognitive impairment; WBC, white blood cell; RBC, red blood cell; TBil, Total bilirubin; ALT, Alanine transaminase; BUN, Blood urea nitrogen; TC,total cholesterol; TG, triglyceride; HDL-C, high-density lipoprotein cholesterol; GLU, Glucose.

**Supplementary Table 3. Other Lifestyle Factors in Later Life between MCI Elders and Controls [n (%)]**

| **Variables** | **Control**  **N=658**  **n (%)** | **MCI**  **N=131**  **n (%)** | ***χ^2^*** | ***P*** |
| --- | --- | --- | --- | --- |
| **Smoking** |  |  | 0.009 | .924 |
| Yes | 133 (20.2) | 26 (19.8) |  |  |
| No | 525 (79.8) | 105 (80.2) |  |  |
| **Drinking*** |  |  | 0.037 | .848 |
| Yes | 120 (18.3) | 23 (17.6) |  |  |
| No | 537 (81.7) | 108 (82.4) |  |  |
| **How often participate in mental activities** |  |  | 1.146 | .284 |
| ≤3 per week | 515 (78.3) | 108 (82.4) |  |  |
| ≥4 per week | 143 (21.7) | 23 (17.6) |  |  |
| **How often watch TV alone or listen to the radio*** |  |  | 0.190 | .663 |
| ≤3 per week | 122 (18.5) | 22 (16.9) |  |  |
| ≥4 per week | 536 (81.5) | 108 (83.1) |  |  |
| **Feeling sleepy during the day** |  |  | 0.752 | .386 |
| ≤3 per week | 586 (89.1) | 120 (91.6) |  |  |
| ≥4 per week | 72 (10.9) | 11 (8.4) |  |  |
| **The sleep time** |  |  | 0.169 | .681 |
| <9 h/night | 581 (88.3) | 114 (87.0) |  |  |
| ≥9 h/night | 77 (11.7) | 17 (13.0) |  |  |
| **Post-lunch nap** |  |  | 1.132 | .287 |
| Yes | 501 (76.1) | 94 (71.8) |  |  |
| No | 157 (23.9) | 37 (28.2) |  |  |

* Due to the missing values in some variables, the total numbers may not equal to 658 or 131.

MCI, mild cognitive impairment.

**Supplementary Table 4. Other Dietary Habits between MCI and Controls E**lders

| **Variables** | **Control**  **N=658**  **n (%)** | **MCI**  **N=131**  **n (%)** | ***χ^2^*** | ***P*** |
| --- | --- | --- | --- | --- |
| **How often consume meat and meat products** |  |  | 0.528 | .467 |
| ≤3 per week | 26 (4.0) | 7 (5.3) |  |  |
| ≥4 per week | 632 (96.0) | 124 (94.7) |  |  |
| **How often consume milk and dairy products** |  |  | 0.249 | .618 |
| ≤3 per week | 417 (63.4) | 80 (61.1) |  |  |
| ≥4 per week | 241 (36.6) | 51 (38.9) |  |  |
| **How often consume legumes** |  |  | 0.970 | .325 |
| ≤3 per week | 546 (83.0) | 104 (79.4) |  |  |
| ≥4 per week | 112 (17.0) | 27 (20.6) |  |  |
| **How often consume nuts** |  |  | 0.257 | .612 |
| ≤3 per week | 559 (85.0) | 109 (83.2) |  |  |
| ≥4 per week | 99 (15.0) | 22 (16.8) |  |  |
| **How often consume eggs** |  |  | 1.428 | .232 |
| ≤3 per week | 344 (52.3) | 61 (46.6) |  |  |
| ≥4 per week | 314 (47.7) | 70 (53.4) |  |  |
| **How often consume seafood** |  |  | 2.196 | .138 |
| ≤3 per week | 632 (96.0) | 122 (93.1) |  |  |
| ≥4 per week | 26 (4.0) | 9 (6.9) |  |  |
| **How often consume seaweed** |  |  | 0.344 | .558 |
| ≤3 per week | 639 (97.1) | 129 (98.5) |  |  |
| ≥4 per week | 19 (2.9) | 2 (1.5) |  |  |
| **How often consume mushroom** |  |  | 0.022 | .882 |
| ≤3 per week | 620 (94.2) | 123 (93.9) |  |  |
| ≥4 per week | 38 (5.8) | 8 (6.1) |  |  |
| **How often consume garlic** |  |  | 0.126 | .723 |
| ≤3 per week | 257 (39.1) | 49 (37.4) |  |  |
| ≥4 per week | 401 (60.9) | 82 (62.6) |  |  |
| **How often consume pickled vegetables** |  |  | 0.930 | .335 |
| ≤3 per week | 607 (92.2) | 124 (94.7) |  |  |
| ≥4 per week | 51 (7.8) | 7 (5.3) |  |  |
| **How often consume fried food** |  |  | 1.661 | .197 |
| ≤3 per week | 645 (98.0) | 126 (96.2) |  |  |
| ≥4 per week | 13 (2.0) | 5 (3.8) |  |  |
| **How often consume tea** |  |  | 1.998 | .157 |
| ≤3 per week | 539 (81.9) | 114 (87.0) |  |  |
| ≥4 per week | 119 (18.1) | 17 (13.0) |  |  |
| **How often consume coffee** |  |  | <0.001 | 1.000 |
| ≤3 per week | 646 (98.2) | 129 (98.5) |  |  |
| ≥4 per week | 12 (1.8) | 2 (1.5) |  |  |
| **How often consume vitamins** |  |  | 1.024 | .312 |
| ≤3 per week | 601 (91.3) | 116 (88.5) |  |  |
| ≥4 per week | 57 (8.7) | 15 (11.5) |  |  |
| **How often consume trace element supplement such as Calcium, zinc etc.** |  |  | 0.278 | .598 |
| ≤3 per week | 493 (74.9) | 101 (77.1) |  |  |
| ≥4 per week | 165 (25.1) | 30 (22.9) |  |  |
| **How often consume herbal supplements** |  |  | 0.006 | .937 |
| ≤3 per week | 619 (94.1) | 123 (93.9) |  |  |
| ≥4 per week | 39 (5.9) | 8 (6.1) |  |  |
| **How often consume fish oil and other supplements containing unsaturated fatty acids** |  |  | 0.706 | .401 |
| ≤3 per week | 629 (95.6) | 123 (93.9) |  |  |
| ≥4 per week | 29 (4.4) | 8 (6.1) |  |  |

MCI, mild cognitive impairment.
